# P-1998. A new normal? Dynamic patterns of healthcare engagement during the COVID-19 pandemic among people with HIV

**DOI:** 10.1093/ofid/ofae631.2155

**Published:** 2025-01-29

**Authors:** Jasmine Manalel, Jennifer Kaufman, Yiyi Wu, Alvin Gao, Carey Brandenburg, Luis Scaccabarrozzi, Vera Antonios, Jerome Ernst, Mark Brennan-Ing

**Affiliations:** Hunter College, New York, New York; Hunter College, New York, New York; Hunter College, New York, New York; Hunter College, New York, New York; Amida Care, New York, New York; Amida Care, New York, New York; Amida Care, New York, New York; Amida Care, New York, New York; Hunter College, The City University of New York, New York, NY

## Abstract

**Background:**

People with HIV (PWH) require regular and consistent engagement with the healthcare system to maintain viral suppression. This includes regular visits to primary care (PCP) and other healthcare providers. Access to behavioral health care for PWH is also critical given the association of behavioral health medication adherence. PWH were at increased risk of experiencing COVID-19 pandemic-related disruptions to their care due to pandemic mitigation measures. We examined PCP and behavioral health visits among PWH to assess dynamic patterns of healthcare engagement before and during the COVID-19 pandemic.

**Figure:**
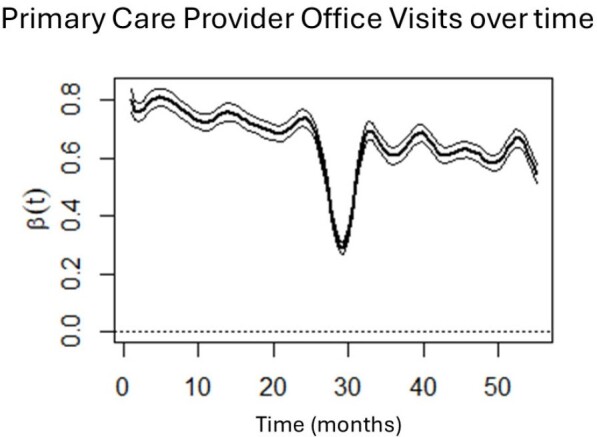

PCP office visits from January 2018 (Month 1) to July 2022 (Month 55). Month 26 = March 2020, start of COVID-19 pandemic in USA.

**Methods:**

Data came from the health claims and clinical records of 3,292 PWH aged 18 to 65 who were continuously enrolled in a Medicaid managed care plan based in New York City from January 2018 through July 2022. Most of the sample was over age 46 (61%), identified as cisgender male (62%), and non-Hispanic Black (66%). The number of PCP and behavioral health visits, including office and telehealth, was summed by month. Telehealth utilization was reported beginning in February 2020 (8.6% and 21.9% of PCP and behavioral health visits, respectively). We estimated intercept-only time varying estimation models (TVEM) to examine dynamic patterns of PCP and behavioral health utilization.

**Figure:**
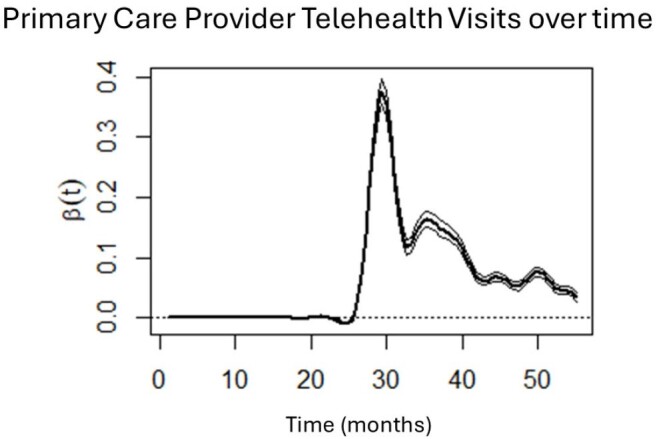

PCP telehealth visits from January 2018 (Month 1) to July 2022 (Month 55). Month 26 = March 2020, start of COVID-19 pandemic in USA.

**Results:**

Results showed that PCP office visits were stable pre-pandemic, decreased sharply at the start of the pandemic, and gradually increased, but never reached pre-pandemic levels or stability. Behavioral health visits mirrored this trajectory. Sharp increases in telehealth utilization were observed around March 2020, for both PCP and behavioral health visits, with both decreasing as the pandemic progressed. Importantly, telehealth visits do not appear make up to completely and consistently for lower office visits during the pandemic.

**Figure:**
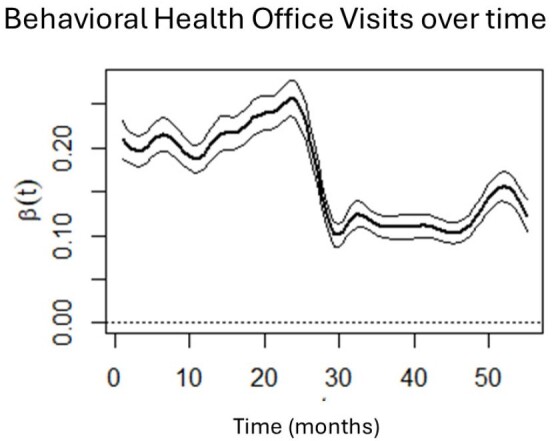

Behavioral health office visits from January 2018 (Month 1) to July 2022 (Month 55). Month 26 = March 2020, start of COVID-19 pandemic in USA.

**Conclusion:**

While the patterns of PCP, behavioral health, and telehealth utilization may correspond to various phases of the pandemic, it is not clear if decreasing utilization is due to care disengagement or ongoing disruptions to health care provision. The persistent decrease in PCP and behavioral health visits may interfere with public health goals regarding ending the HIV epidemic and U.N. 95-95-95 targets.

**Figure:**
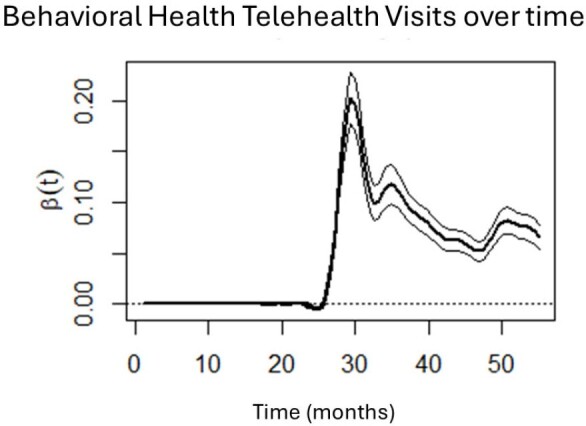

Behavioral health telehealth visits from January 2018 (Month 1) to July 2022 (Month 55). Month 26 = March 2020, start of COVID-19 pandemic in USA.

**Disclosures:**

Jasmine Manalel, PhD, Gilead Sciences: Grant/Research Support Jennifer Kaufman, MPH, Gilead Sciences: Grant/Research Support Yiyi Wu, MA, Gilead Sciences: Grant/Research Support Alvin Gao, n/a, Gilead Sciences: Grant/Research Support Carey Brandenburg, n/a, Gilead Sciences: Grant/Research Support Luis Scaccabarrozzi, MPH, Gilead Sciences: Grant/Research Support Vera Antonios, MD, Gilead Sciences: Grant/Research Support Jerome Ernst, MD, Gilead Sciences: Grant/Research Support Mark Brennan-Ing, PhD, Gilead Sciences: Grant/Research Support

